# Comparative evolutionary history of two closely related desert plant, *Convolvulus tragacanthoide* and *Convolvulus gortschakovii* (Convolvulaceae) from northwest China

**DOI:** 10.1002/ece3.9355

**Published:** 2022-09-20

**Authors:** Shuwen Jia, Lina Xu, Xiaoxiao Geng, Hongxiang Zhang

**Affiliations:** ^1^ Hainan Academy of Ocean and Fisheries Sciences Haikou China; ^2^ CAS Center for Excellence in Molecular Plant Science Shanghai China; ^3^ Key Laboratory of Utilization and Conservation for Tropical Marine Bioresources Hainan Tropical Ocean University Sanya China; ^4^ State Key Laboratory of Desert and Oasis Ecology Xinjiang Institute of Ecology and Geography, Chinese Academy of Sciences Urumqi China; ^5^ The Specimen Museum of Xinjiang Institute of Ecology and Geography Chinese Academy of Sciences Urumqi China

**Keywords:** 200 mm precipitation line, climate change, desert plants, Phylogeographic structure, population dynamics

## Abstract

Desert ecosystems are one of the most fragile ecosystems on Earth. The study of the effects of paleoclimatic and geological changes on genetic diversity, genetic structure, and species differentiation of desert plants is not only helpful in understanding the strategies of adaptation of plants to arid habitats, but can also provide reference for the protection and restoration of vegetation in desert ecosystem. Northwest China is an important part of arid regions in the northern hemisphere. *Convolvulus tragacanthoides* and *Convolvulus gortschakovii* are closely related and have similar morphology. Through our field investigation, we found that the annual precipitation of the two species distribution areas is significantly different. Thus, *C*. *tragacanthoides* and *C. gortschakovii* provide an ideal comparative template to investigate the evolutionary processes of closely related species, which have adapted to different niches in response to changes in paleogeography and paleoclimate in northwest China. In this study, we employed phylogeographical approaches (two cpDNA spacers: *rpl14–rpl36* and *trnT–trnY*) and species distribution models to trace the demographic history of *C. tragacanthoides* and *C. gortschakovii*, two common subshrubs and small shrubs in northwest China. The results showed the following: (1) Populations of *C. tragacanthoides* in northwest China were divided into three groups: Tianshan Mountains—Ili Valley, west Yin Mountains—Helan Mountains‐Qinglian Mountains, and Qinling Mountains—east Yin Mountains. There was a strong correlation between the distribution of haplotypes and the floristic subkingdom. The three groups corresponded to the Eurasian forest subkingdom, Asian desert flora subkingdom, and Sino‐Japanese floristic regions, respectively. Thus, environmental differences among different flora may lead to the genetic differentiation of *C. tragacanthoides* in China. (2) The west Yin Mountains—Helan Mountains‐Qinglian Mountains, and Qinling Mountains—east Yin Mountains were thought to form the ancestral distribution range of *C. tragacanthoides.* (3) *C. tragacanthoides* and *C. gortschakovii* adopted different strategies to cope with the Pleistocene glacial cycle. *Convolvulus tragacanthoides* contracted to the south during the glacial period and expanded to the north during the interglacial period; and there was no obvious north–south expansion or contraction of *C. gortschakovii* during the glacial cycle. (4) The interspecific variation of *C*. *tragacanthoides* and *C. gortschakovii* was related to the orogeny in northwest China caused by the uplift of the Tibetan Plateau during Miocene. (5) The 200 mm precipitation line formed the dividing line between the niches occupied by *C*. *tragacanthoides* and *C. gortschakovii*, respectively. In this study, from the perspective of precipitation, the impact of the formation of the summer monsoon limit line on species divergence and speciation is reported, which provides a new perspective for studying the response mechanism of species to the formation of the summer monsoon line, and also provides a clue for predicting how desert plants respond to future environmental changes.

## Graphical abstract

In this study, we employed phylogeographical approaches and species distribution models (SDMs) to trace the demographic history of *Convolvulus tragacanthoides* and *Convolvulus gortschakovii*, two subshrubs adapted to arid and semi‐arid climate, respectively. Our results show that the long‐term climatic differences between the arid and semi‐arid areas might increase species divergence and local adaptation.

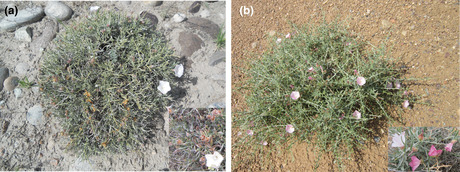

## 1 INTRODUCTION

Climate changes have posed major challenges for biodiversity conservation (Heller & Zavaleta, 2009; Meng et al., 2021). To effectively ensure the persistence of biodiversity under the threats of climate change, it is vital to accurately determine its effects on species distribution. The effects of the Pleistocene glacial–interglacial cycle strongly influence the geographical distribution and genetic diversity of global species, having resulted in local species‐level extinctions, migrations, and induced allopatric speciation (Willis & Niklas, 2004). For instance, many temperate species distributions contracted at southerly refugia during glacial periods and expanded back into northern regions during interglacial periods in Europe (Hewitt, 2000). Similarly, many desert plants' distributions in northwest China receded to relatively moist refugia during the dry glacial period, subsequently expanding during the relatively moist interglacial period (Gao et al., 2014; Jia & Zhang, 2019; Ma et al., 2012; Su et al., 2016; Zhang et al., 2020). Geological changes since the Miocene have also had important effects on plant distribution. For example, previous phylogeographic studies have indicated that environmental changes caused by the uplift of the Tibetan Plateau have led to the differentiation of desert plant populations in northwest China (Jia & Zhang, 2021; Liu et al., 2006; Meng et al., 2015). Thus, understanding the impacts of climate change and geological processes on the spatial distribution of plants, especially vulnerable desert plants, can not only provide insight into the history of population dynamics, but can also provide a source of data for the mitigation of future climatic and environmental changes.

Central Asia comprises by majority arid and semi‐arid zones. The interaction between topographic structure and climatic conditions forms a more complex landscape pattern in the region. The arid region of northwest China is located in Central Asia. The vegetation of this region is characterized through adaptations to drought resistance and infertility tolerance, which have been advantageous for maintaining and restoring desert vegetative ecosystem. Therefore, phylogeographic patterns and the evolutionary history of species in this region have attracted the attention of an increasing number of biologists. Many studies have shown that climate change and orogeny from the Miocene to Pleistocene have contributed greatly to the patterns of genetic diversity among desert plants (Jia et al., 2016; Jia & Zhang, 2019; Ma et al., 2012). The relatively humid semi‐arid region provided refugia for desert plants during the glacial period and further provided a dispersal corridor during the interglacial period, which has had an important impact on the evolution of desert plants and the formation of lineages (Jia & Zhang, 2021; Shi & Zhang, 2015; Zhang & Zhang, 2012). Northwest China is a very vast region, including a variety of landforms and flora. Understanding the driving factors and mechanisms of species diversity and persistence in this region is of great significance for biogeography, evolutionary biology, and conservation biology in the global arid area.

**FIGURE 1:**
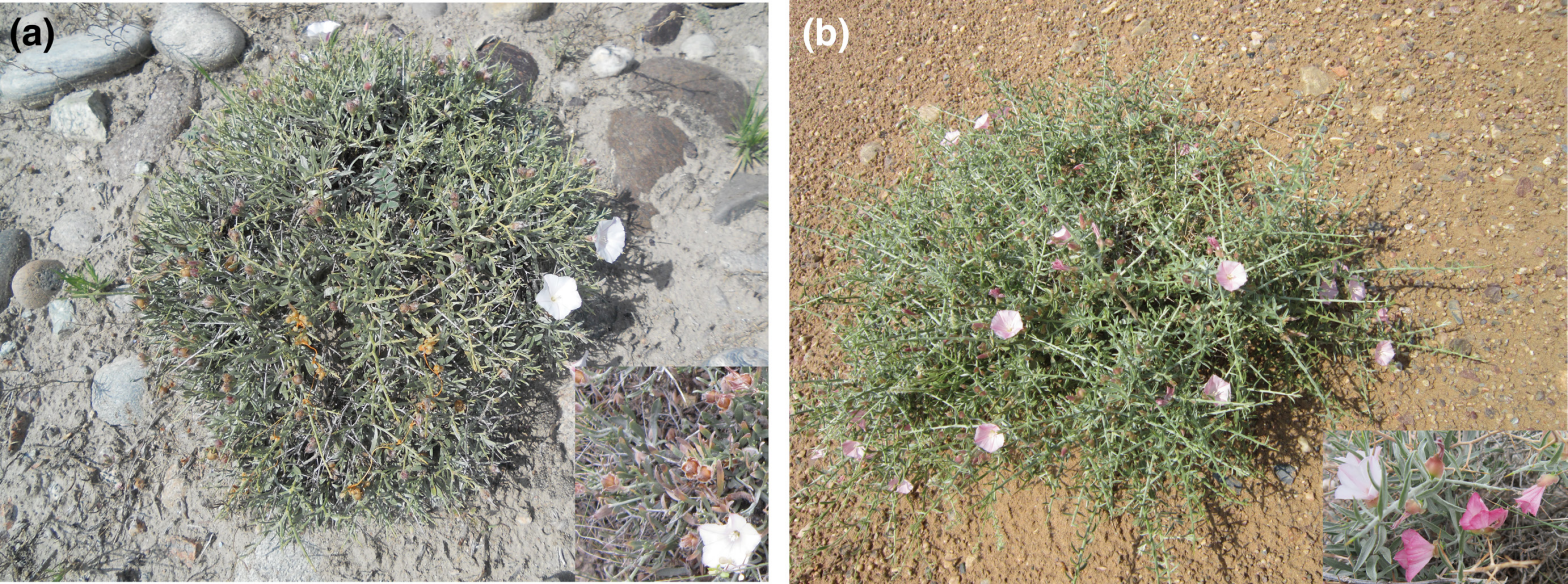
Morphological characteristics of *Convolvulus tragacanthoide* and *Convolvulus gortschakovii* in Northwest China. (a) *C. tragacanthoide*. (b) *C. gortschakovii*.

**FIGURE 2:**
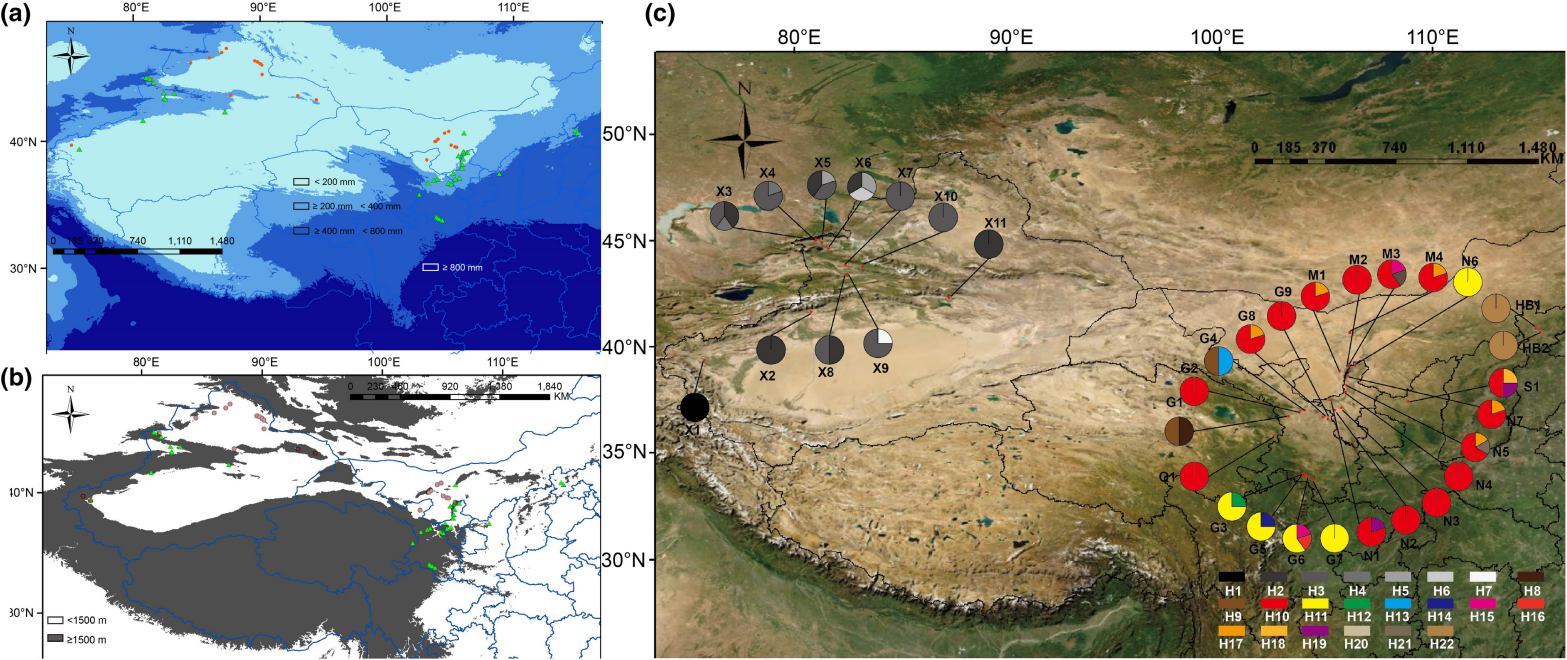
Distribution of sampling points of *Convolvulus tragacanthoides* and *Convolvulus gortschakovii*. (a) Difference of annual precipitation between *C. tragacanthoide* and *C. gortschakovii* distribution points. (b) Altitude difference of *C. tragacanthoide* and *C. gortschakovii* distribution points. (c) Geographic distribution of 22 haplotypes of *C. tragacanthoides* detected in northwest China. In a and b: green triangle: *C. tragacanthoides*. Orange circle: *C. gortschakovii*. The detailed information of the population IDs of *C. tragacanthoides* is in Table 1.

*Convolvulus* (Convolvulaceae) is widely distributed among the temperate and subtropical regions (Wood et al., 2015). According to the chloroplast markers *matK* and *rbcL*, the *Convolvulus* species in Eurasia are diffused from the Mediterranean and the Middle East (Mitchell et al., 2016). Eight species are distributed in China. According to morphological data, five species of *Convolvulus* (*Convolvulus ammannii*, *C. tragacanthoides*, *C. fruticosus*, *C. gortschakovii*, and *C. lineatus*), which are distributed in China, belong to old world undershrubs with sericeal leaves (Wood et al., 2015). *Convolvulus ammannii* and *C. lineatus* are perennial herbs, whereas *C. tragacanthoides*, *C. fruticosus*, and *C. gortschakovii* are subshrubs or small shrubs. *Convolvulus tragacanthoides* and *C. gortschakovii* are mainly distributed in Central Asia, whereas *C. fruticosus* is mainly distributed in Central and Western Asia (Wood et al., 2015). In addition, *C. tragacanthoides* and *C. gortschakovii* are widely distributed throughout northwest China and have similar morphologies, while *C. fruticosus* has a narrow distribution in China. Morphologically, the obvious difference between *C. tragacanthoides* and *C. gortschakovii* is that the two outer sepals of *C*. *gortschakovii* are significantly wider than the three inner sepals, whereas the shape of the inner and outer sepals of *C*. *tragacanthoides* is similar (Figure 1; Wu et al., 2004; Wood et al., 2015). According to Jia and Zhang (2021), except for *C. ammannii*, the evolutionary relationship between *C. tragacanthoides* and *C. gortschakovii* is recent. In our field investigation, we found that the distribution of *C. ammannii* broadly overlapped with that of *C. tragacanthoides* and *C. gortschakovii*, but the distribution of *C. tragacanthoides* and *C. gortschakovii* barely overlapped. Most populations of *C. gortschakovii* were mainly distributed in areas with an annual average precipitation of <200 mm, whereas populations of *C. tragacanthoides* were mainly distributed in areas with an annual average precipitation of >200 mm (Figure 2a), indicating a distribution gradient regulated by precipitation. In addition, compared with *C. gortschakovii*, *C*. *tragacanthoides* is found at much high altitudes, mostly at heights of >1500 m (Figure 2b). The humidification of arid area in northwest China is altitudinal dependent. Under the influence of atmospheric circulation system, precipitation increases with elevation under the action of topographic uplift, which has been proved in the Tianshan Mountains and Qilian Mountains (Yao et al., 2016, 2018; Zhang et al., 2008). Therefore, we assumed that the two species occupy different ecological niches separated by precipitation. Thus, *C*. *tragacanthoides* and *C. gortschakovii* provide an ideal comparative template to investigate the evolutionary processes of closely related species, which have adapted to different niches in response to changes in paleogeography and paleoclimate in northwest China.

Jia and Zhang (2021) used two plastid regions (*rpl14–rpl36* and *trnT–trnY* intron) and the nuclear ribosomal internal transcribed spacer (ITS) region to analyze 25 populations of *C. gortschakovii* from northwest China, employing a phylogeographical approach. Populations of *C. gortschakovii* were divided into three groups: the Junggar Basin, the Alatau Mountains—central Tianshan Mountains—Tarim Basin, and the Alxa Desert. The Tianshan Mountains are considered to pose as a substantial geographical barrier to gene flow between the Junggar Basin and the Tarim Basin, while the former is considered the ancestral distribution area of *C. gortschakovii*. Further, it has been inferred that the Eurasian forest subkingdom plays an important role in the promotion of genetic diversity of xerophytes in the Asian desert flora subkingdom.

In the present study, we conducted a phylogeographical investigation using two plastid regions (*rpl14–rpl36* and *trnT–trnY* intron) and a species distribution model to trace the demographic history of *C*. *tragacanthoides* and *C. gortschakovii* and to discuss any potential phylogeographical events corresponding to paleoclimatic and paleogeographic changes, which may have affected these species. Specifically, we aimed to: (1) compare the genetic diversity, genetic structure, and phylogeographic pattern of two related species and; (2) compare the responses of plants adapted to different degrees of drought to paleoclimate change. These data will provide new insight into historical vegetation dynamics and plant species evolution in northwest China.

## 2 MATERIALS AND METHODS

### 2.1 Sampling, DNA extraction, amplification, and sequencing

We sampled 164 individuals from 35 populations of *C. tragacanthoides*, covering almost the entire distribution range of the species in China between June and August 2015. In each population, 2–6 individuals were collected, and fresh leaves were rapidly dried in silica gel. The coordinates of each population were recorded using a global positioning system (GPS) unit (Table 1; Figure 2, Figure S1). All silica gel‐dried materials and voucher specimens were stored in state key laboratory of desert and oasis ecology of Xinjiang institute of ecology and geography of Chinese academy of sciences.

**TABLE 1:** Details of sample locations and genetic diversity for *Convolvulus tragacanthoides* populations

Population ID	Latitude	Longitude	Haplotypes	Number	*h* ± SD	*π* ± SD
X1	39.380028	75.7538	H1(4)	4	0	0
X2	41.652500	80.7799	H2(4)	4	0	0
X3	45.008483	80.9842	H2(2),H3(2),H4(1)	5	0.8000 ± 0.1640	0.000535 ± 0.000498
X4	45.011579	81.0294	H3(4),H4(1)	5	0.4000 ± 0.2373	0.000214 ± 0.000272
X5	44.92918	81.3076	H2(2),H3(2),H5(1)	5	0.8000 ± 0.1640	0.000748 ± 0.000637
X6	44.62123	81.6188	H2(2),H5(2),H6(2)	6	0.8000 ± 0.1217	0.000856 ± 0.000677
X7	43.833569	82.401	H3(5)	5	0	0
X8	43.42159	82.4839	H2(2),H3(2)	4	0.6667 ± 0.2041	0.000357 ± 0.000400
X9	43.3421	82.5197	H3(3),H7(1)	4	0.5000 ± 0.2652	0.000535 ± 0.000530
X10	43.777445	83.2344	H3(6)	6	0	0
X11	42.325460	87.2506	H2(5)	5	0	0
G1	36.724373	103.238	H8(1),H9(1)	2	1.0000 ± 0.5000	0.004280 ± 0.004540
G2	36.934144	103.749	H10(4)	4	0	0
G3	34.046344	103.905	H11(3),H12(1)	4	0.5000 ± 0.2652	0.000268 ± 0.000333
G4	37.019112	103.981	H9(2),H13(2),	4	0.6667 ± 0.2041	0.002854 ± 0.002078
G5	33.948645	104.005	H11(3),H14(1)	4	0.5000 ± 0.2652	0.000268 ± 0.000333
G6	33.87824	104.138	H11(3),H15(1),H16(1)	5	0.7000 ± 0.2184	0.001499 ± 0.001107
G7	33.761201	104.394	H11(5)	5	0	0
G8	36.716421	104.856	H10(4),H17(1)	5	0.4000 ± 0.2373	0.000430 ± 0.000428
G9	36.63278	105.084	H10(5)	5	0	0
S1	37.445703	108.886	H10(2),H18(1),H19(1)	4	0.8333 ± 0.2224	0.000895 ± 0.000780
N1	37.466909	105.235	H10(4),H19(1)	5	0.4000 ± 0.2373	0.000215 ± 0.000273
N2	37.015058	105.397	H10(4)	4	0	0
N3	37.11945	105.734	H10(5)	5	0	0
N4	37.887863	105.922	H10(5)	5	0	0
N5	38.18359	105.948	H10(4),H17(1),H20(1)	6	0.6000 ± 0.2152	0.000752 ± 0.000614
N6	39.116128	106.394	H11(5)	5	0	0
N7	38.618741	106.022	H10(4),H17(1)	5	0.4000 ± 0.2373	0.000430 ± 0.000428
M1	38.872545	105.66	H10(4),H17(1)	5	0.4000 ± 0.2373	0.000430 ± 0.000428
M2	39.078692	106.043	H10(5)	5	0	0
M3	39.186725	106.062	H10(3),H15(1),H21(1)	5	0.7000 ± 0.2184	0.001074 ± 0.000843
M4	40.653684	106.105	H10(4),H17(1)	5	0.4000 ± 0.2373	0.000430 ± 0.000428
Q1	35.793424	102.553	H10(4)	4	0	0
HB1	40.845115	114.881	H22(5)	5	0	0
HB2	40.677966	115.072	H22(5)	5	0	0

Abbreviations: X, Xinjiang Province; G, Gansu Province; S, Shanxi; N, Ninxia Province; M, Inner Mongolia Province; Q, Qinghai Province; HB, Hebei Province; *h*, haplotype diversity; π, nucleotide diversity.

Total genomic DNA was extracted from silica gel‐dried leaves using a modified CTAB protocol, as described by Doyle and Doyle (1987). Two primer pairs *rpl14–rpl36* (Forward: AAGGAAATCCAAAAGGAACTCG; Reverse: GGRTTGGAACAAATTACTATAATTCG; Shaw et al., 2007) and *trnT–trnY* (Forward; CTACCACTGAGTTAAAAGGG; Reverse: CCGAGCTGGATTTGAACCA; Shaw et al., 2005) were used to amplify the *rpl14–rpl36* region and the *trnT–trnY* intron, respectively. The PCR conditions implemented for these regions were 95°C for 2 min, followed by 30 cycles of 95°C for 30 s, annealing at 52°C for 30 s, 72°C for 45 s, and an additional extension at 72°C for 5 min. PCR products were sequenced using an ABI 3730 automated sequencer (Shanghai Bioengineering, Shanghai, China). Sequences were edited in Seqman (Lasergene, DNASTAR Inc., Madison, WI, USA), aligned using CLUSTAL_W (Thompson et al., 1994), and manually adjusted where necessary.

The data of GPS coordinates and sequence for *C. gortschakovii* (accession numbers: MN161812 ‐ MN161835) were obtained from Jia and Zhang (2021).

### 2.2 Data analysis of *C. tragacanthoides*

The two cpDNA fragments were combined and treated as haplotypes (Uribe‐Convers & Tank, 2015). Haplotypes were identified using DnaSP 6 (Rozas et al., 2017). The haplotype diversity (*h*) and nucleotide diversity (*π*) of each population were calculated using Arlequin v.3.11 (Excoffier et al., 2005). The average genetic diversity within populations (*H*_S_) and total genetic diversity (*H*_T_) were calculated using PERMUT v.1.0 (Pons & Petit, 1996).

Phylogeographical signals were inferred by testing for *N*_ST_ and *G*_ST_ using PERMUT v. 1.0 (Pons & Petit, 1996). SAMOVA v. 1.0 was used for population subdivision analysis (Dupanloup et al., 2002). The program was run for 10,000 iterations in the range 2 ≤ *K* ≤ 10. A Bayesian cluster analysis was used to assess the genetic structuring of populations of *C. tragacanthoides* using STRUCTURE v. 2.3.4 (Pritchard et al., 2000). To obtain stable results, the program was run five times independently, the number of iterations and the burn‐in were 20,000 and 120,000, respectively, and the genetic packet number (*k*) was set from 2 to 10. The DeltaK method was used in the Structure Harvester online tool to determine the optimal number of groups. A molecular variance analysis (AMOVA) was performed by Arlequin v. 3.11 (Excoffier et al., 2005) to assess the level of genetic differentiation between geographic groups and populations. A mismatch distribution analysis (MDA), Tajima's *D* test and Fu's *F*_S_ test were conducted in Arlequin v. 3.11 (Excoffier et al., 2005) to determine whether *C. tragacanthoides* was underwent population expansion.

The biogeographic history of *C. tragacanthoides* was reconstructed using statistical dispersal vicariance analysis (S‐DIVA; Yu et al., 2010), Bayesian binary method (BBM; Ronquist & Huelsenbeck, 2003), Lagrange (maximum‐likelihood dispersal–extinction–cladogenesis model, DEC; Ree & Smith, 2008) and Bayes‐Lagrange (S‐DEC; Beaulieu et al., 2013) as implemented in RASP v. 3.2 (Yu et al., 2015). Geographic regions were based on the distribution characteristics of the population and haplotypes. We considered species to be distributed among four areas: (A) Tianshan Mountains and Ili Valley belong to the Eurasian forest subkingdom, and the haplotype is closely related in analysis of network, including X1, X2, X3, X4, X5, X6, X7, X8, X9, X10, and X11; (B) Qilian Mountains, Helan Mountains, and west Yin Mountain belong to the Asian desert flora subkingdom, and the haplotypes are closely related, including Q1, G1, G2, G4, G8, G9, M1, M2, M3, M4, N1, N2, N3, N4, N5, N6, N7, and Q1; (C) Qinling Mountains belong to the Sino‐Japanese floristic regions, and the haplotypes are closely related, including G3, G5, G6, and G7; (D) east Yin Mountains, which is distant from other distribution areas and comprises the same haplotype, including HB1 and HB2. Since S1 is not in the main mountain range and has fewer distributed communities, this distribution was not considered. The maximum clade credibility (MCC) trees of these analyses were obtained from BEAST v. 1.8.0 (Drummond et al., 2012) using the combined cpDNA data set. We used RASP v. 3.2 (Yu et al., 2015) on a posterior distribution of the last 1000 trees (in front of the 199,008 trees, which were discarded by RASP v. 3.2) from the cpDNA BEAST analyses. According to the guide of RASP v. 3.2, the distributions of outgroups might limit the biogeographic analysis; thus, the outgroup sequences were excluded from the analysis.

### 2.3 Combined analysis of *C. tragacanthoides* and *C. gortschakovii*

The level of gene flow between *C. tragacanthoides* and *C. gortschakovii* was using DnaSP 6 (Rozas et al., 2017). Genealogical relationships among haplotypes of *C. tragacanthoides* and *C. gortschakovii* were described in Network v. 4.6.1.3, followed by the median Joining algorithm (Bandelt et al., 1999). A Bayesian cluster analysis was used to assess the genetic structure of populations of *C. tragacanthoides* and *C. gortschakovii* using STRUCTURE v. 2.3.4 software (Pritchard et al., 2000). The parameter settings are the same as those described previously. The divergence time between *C. tragacanthoides* and *C. gortschakovii* was investigated using BEAST v. 1.8.0 (Drummond et al., 2012). An uncorrelated lognormal relaxed clock model and Yule prior were used in this analysis. The GTR + G + I substitution model was selected using Akaike information criterion (AIC) values as implemented in MEGA6 (Tamura et al., 2013) for use in this analysis. The Markov chain Monte Carlo (MCMC) analyses were run for 200, 000, 000 generations, sampling every 1000 generations with a 10% initial burn‐in. The effective sample size (ESSs >200) was determined using Tracer 1.7 (Rambaut et al., 2018), and the final tree was summarized and annotated in FigTree v1.4.0 (http://tree.bio.ed.ac.uk/software/figtree/). In this case, three fossil calibration points were used according to Eserman et al. (2014). A single fossilized pollen grain of *Calystegiapollis microechinatus* was used to calibrate the root of the family Convolvulaceae between 47.8 and 56.0 Ma (millions of years ago) (Muller, 1981). Three Merremia Dennst. ex Endl. fossilized pollen grains were used to calibrate the roots of two Merremieae species (*Operculina macrocarpa* (L.) Urb., *Merremia hederacea* (Burm. f.) Hall. f.) between 41.2 and 47.8 Ma (Legoux, 1978; Muller, 1981; Pares Regali et al., 1974a, 1974b). A solanum‐like fossilized pollen grain was used to calibrate the roots of the Nicotianoideae and Solanoideae clades of Solanaceae species between 23 and 33.9 Ma (Graham, 2010; Martínez‐Hernández & Ramírez‐Arriaga, 1999). The three nodes calibrated with fossilized pollen grains assumed a normal distribution (Convolvulaceae: mean 51.9 Ma, standard deviation 5.8 Ma; *Merremieae* species: mean 44.5 Ma, standard deviation 4.7 Ma; *Solanaceae* species: mean 28.45 Ma, standard deviation 7.7 Ma). The outgroups were similar to that used by Eserman et al. (2014), with slight changes. All outgroup sequences were obtained from GenBank. Species names and GenBank accession numbers of the outgroups are available in Table S1.

### 2.4 Present and past distribution modeling and landscape connectivity

The maximum entropy method was implemented in MAXENT 3.4.4 (Phillips et al., 2020) to simulate the present geographic distribution of *C. tragacanthoides* and *C. gortschakovii*, respectively. Since there are fewer specimen records outside of China than those found within, there exists a lack of accurate coordinates, to accurately predict the niche space occupied by species; this study uses the coordinates collected in the field. The grid data corresponding to the 19 bioclimatic variables were downloaded from the WorldClim database (http://www.worldclim.org/) with a resolution of 2.5 m. The periods involved include the contemporary mid‐Holocene (~6 ka) (ka: thousand years ago) and the Last Glacial Maximum (LGM, ~22 ka), where the climate simulation models for the last two historical periods included the Community Climate System Model 4 (CCSM4). Considering that there may be a strong correlation between these climatic factors, the Pearson correlation coefficient (r) was used to remove climatic factors with a correlation *r* > 0.8 (Tables S2, S3). We used 75% of the sites to train the model and 25% of the sites to test it. All estimates use the median value of 10 ten replicate results, and the areas under the receiver operating characteristic (AUC) were used to evaluate the accuracy of the model. A contribution rate estimation and Jackknife test were used to evaluate the effects of different environmental variables on the geographical distribution of *C. tragacanthoides* and *C. gortschakovii*.

To identify landscape connectivity, the SDMTOOLBOX toolkit (Brown, 2014) was used to convert the species distribution model into a “dispersal cost” layer, combined with the shared chloroplast haplotypes among each population to calculate the sum of all the lowest cost paths, the final result of which was presented in ARCMAP 10.1 (ESRI).

## 3 RESULTS

### 3.1 Analysis of *C. tragacanthoides*

After alignment, the sequence lengths of *rpl14–rpl36* and *trnT–trnY* were 1018 bp and 878 bp, respectively. GenBank accession numbers of the *rpl14–rpl36* and *trnT–trnY* sequences in Table S4. The total length of the sequence after ligation was 1896 bp. After sequence analysis of 164 individuals from 35 populations, 22 chloroplast haplotypes were identified, including 12 SNPs and five insertion/deletion sites. Among them, haplotypes H1–H7 were distributed in the western region and H8–H22 were distributed in the eastern region. In the western region, H3 and H2 were the most common haplotypes and existed in seven and six populations, respectively, while H4 and H5 exist in two populations. In the eastern region, H10 was the most common haplotype and was present in 15 populations. H11 and H17 were less and were distributed among the five populations; H9, H15, H19 and H22 were distributed in two populations. The other 11 haplotypes were private haplotypes and only existed in one population, accounting for 50% of the total haplotypes. Among the 35 populations, 13 contained only one haplotype, accounting for 44.44% of the total population (Figure 2c; Table 1).

There was a large number of populations with only one haplotype, and the geographical distribution pattern of haplotypes resulted in a low average genetic diversity (*H*s = 0.328) and a high total genetic diversity (*H*_T_ = 0.833). In populations with more than three individuals, the highest diversity of haplotypes was observed in population S1 (*h* = 0.8333), and the highest nucleotide diversity was observed in population G6 (*π* = 1.499 × 10^−3^).

The PERMUT analysis showed that the *N*_ST_ (0.882) value of *C. tragacanthoides* was significantly higher than that of the *G*_ST_ (0.607) value, indicative of significant phylogeographic structure.

**FIGURE 3:**
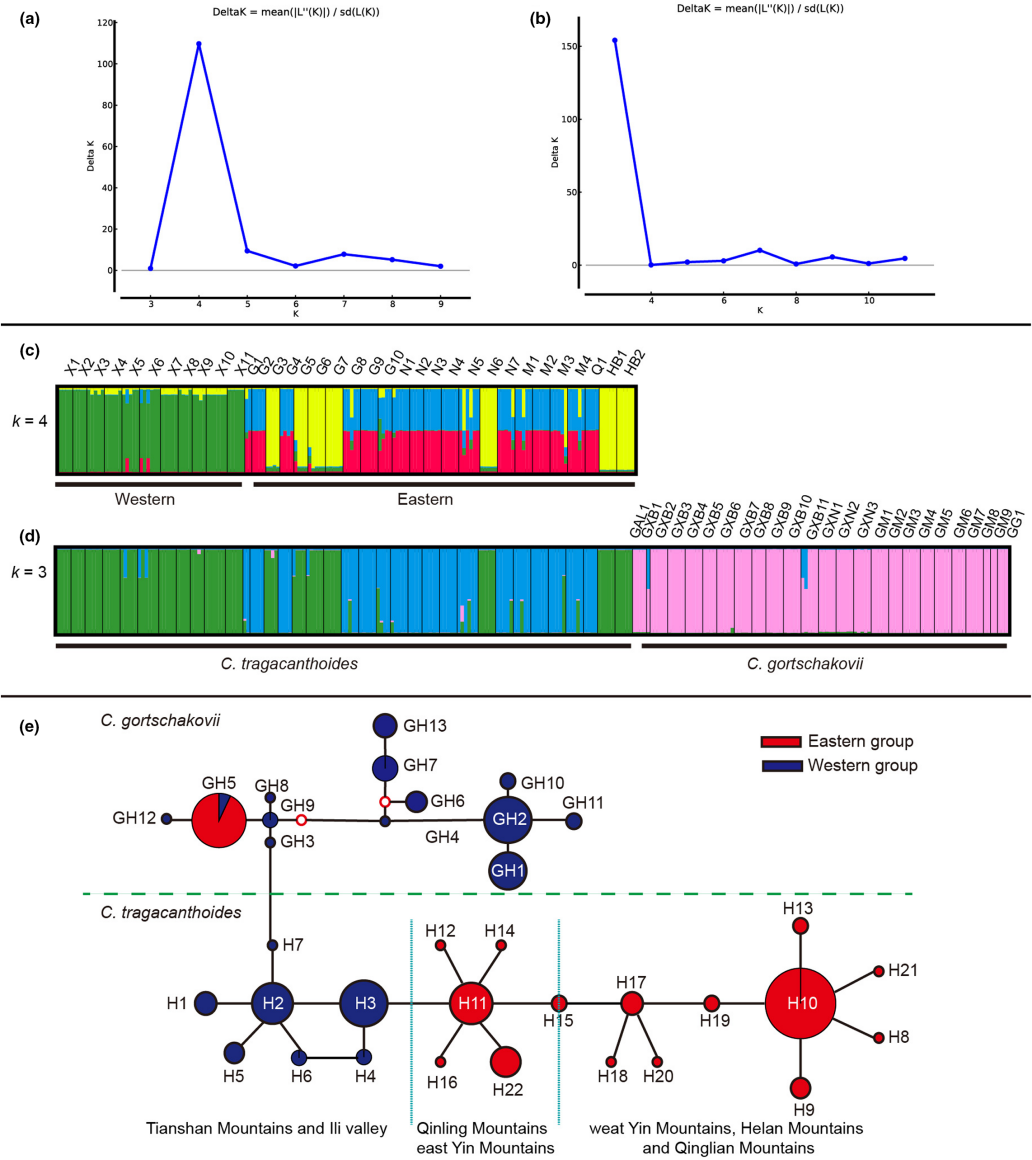
(a) *ΔK* value of STRUCTURE analysis of *Convolvulus tragacanthoides*. (b) The *ΔK* value of STRUCTURE analysis of *C. tragacanthoides* and *Convolvulus gortschakoviis*. (c) Bayesian assignment probability analysis of *C. tragacanthoides*. Four inferred groups were represented by four colors (red, green, blue, and yellow). (d) Bayesian assignment probability analysis of *C. gortschakoviis* and *C. tragacanthoides*. Three inferred groups were represented by three colors (pink, green, and blue). (e) The network found in *C. tragacanthoides* and *C. gortschakovii*. Two red circles indicate missing haplotypes; circle size represents proportional to haplotype frequency.

SAMOVA showed a sharp increase in *F*_CT_ values from *K* = 3 to *K* = 4. When *K* ≥ 4, the grouping structure begins to disappear. Thus, the grouping scheme corresponding to *K* = 3 is as follows: (group 1) populations X1‐11, belonging to the Tianshan Mountains and Ili River Valleys (TI); (group 2) populations G1‐2, G4, G8‐9, S1, N1‐5, N7, M1‐4, Q1, belonging to the west Yin Mountains, Helan Mountains, and Qingling Mountains (YHQ); (group 3) populations G3, G5‐7, N6, HB1‐2, belonging to the Qinling Mountains and east Yin Mountains (QY). A Bayesian cluster analysis showed that the optimal grouping number (*k*) of *C. tragacanthoides* populations was four (Figure 3a, c). The Bayesian cluster of populations was similar to the SAMOVA analysis and was consistent with the geographical distribution of the populations. According to the Bayesian cluster analysis, when the *K* = 4, the Deltak value is the largest. Interestingly, most YHQ individuals contained two genetic lineages, while the individuals of TI and QY contained a single genetic lineage, respectively. The AMOVA analysis showed that there was significant genetic differentiation among the three groups (*F*_CT_ = 0.83613, *P* < .001), and the differentiation level was high, with 83.61% of the genetic variation distributed among the three geographic groups (Table 2).

**TABLE 2:** Results of AMOVA of *Convolvulus tragacanthoides*

Source of variation	df	Sum of squares	Variance components	Percentage of variation	Fixation indices
Among groups	2	465.477	4.45951 Va	83.61	*F*_SC_: 0.57072
Among populations					*F*_ST_: 0.92965
Within groups	32	86.617	0.49882 Vb	9.35	
Within populations	129	48.4	0.37519 Vc	7.03	*F*_CT_: 0.83613
Total	163	600.494	5.33352		

The MDA analysis was used to estimate population dynamics. The observed multiple distribution for all samples and the group level rejected a recent history of population expansion (Figure S2). In contrast, the two test statistics for the selective neutrality of YHQ, QY and Qingling were negative (Table 3), suggesting a sudden population expansion.

**TABLE 3:** Results of neutrality tests for *Convolvulus tragacanthoides*

	Tajima's *D*	*P*‐value	Fu's *F*_S_	*P*‐value
Western	−0.42306	.379	1.82508	.813
Eastern	0.25676	.661	−1.87125	.32
YHQ	−1.00423	.165	−2.87478	.119
QY	−1.13059	.153	−1.40601	.157
Qingling	−1.85306	.008	−1.25673	.121
All	1.00927	.861	0.84821	.647

Abbreviations: YHQ, west Yin Mountains, Helan Mountains, and Qilian Mountains; QY, Qinling Mountains and east Yin Mountains; Qingling, Qingling Mountains.

**FIGURE 4:**
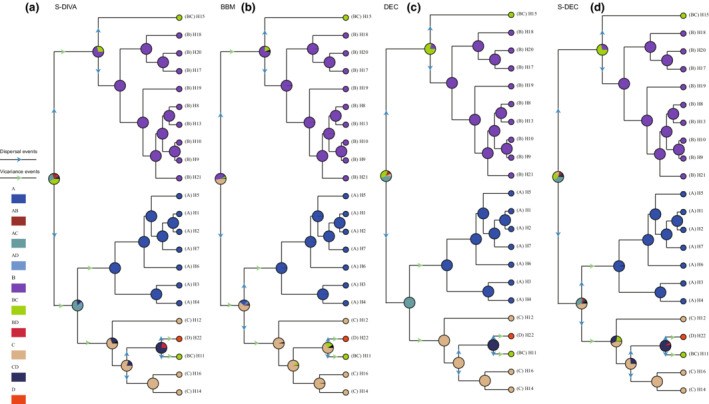
Ancestral area reconstruction of *Convolvulus tragacanthoides* using the Statistical Dispersal Vicariance Analysis (S‐DIVA), Bayesian Binary Method (BBM), Lagrange (maximum‐likelihood dispersal–extinction–cladogenesis model, DEC), and Bayes‐Lagrange (S‐DEC) implemented in RASP. Possible dispersal events are indicated by blue arrow, and vicariance events are indicated by green arrow. (a) Tianshan Mountains and Ili Valley; (b) Qilian Mountains, Helan Mountains, and west Yin Mountain; (c) Qinling Mountains; (d) east Yin Mountains.

In the ancestral area reconstruction analysis, the ancestral area was estimated as AC and BC by S‐DIVA, B and C by BBM, AC and BC by DEC, and AC and BC by S‐DEC. As regions B (Qilian Mountain, Helan Mountain, and Yin Mountain) and C (Qinling Mountain) always occur as ancestral area, they were identified as two ancestral areas. The order of dispersal events supported by all four models was from BC to A (Tianshan Mountains and Ili Valley), B and C; B to C; and C to D (east Yin Mountains). The results showed that the haplotypes in region A are closely related to the haplotypes in region C, the haplotypes in region D come from region C, and there is gene exchange between the regions B and C. Vicariance events occurred between B and C, A and C, C and D (Figure 4).

### 3.2 Combined analysis of *C. tragacanthoides* and *C. gortschakovii*

The total length of the sequence after ligation was 1905 bp. After sequence analysis of 271 individuals from 60 populations, 35 chloroplast haplotypes were identified. The gene flow between *C. tragacanthoides* and *C. gortschakovii* was 0.43. For *C. tragacanthoides* data, the network supported the division of haplotypes into three geographic groups, TI, YHQ, and QY, and four haplotypes were star‐like in the network. The western group included haplotypes of TI, where H2 was found at the center of the network; and H10, H11, and H17 were in the center of the network for the eastern groups. H10 and H17 and their connected haplotypes were mainly distributed in YHQ, while H11 and its connected haplotypes were mainly distributed in the Qinling Mountains, and H22 was distributed in the east Yin Mountains. The network suggested that the two species were resolved as monophyletic. For *C. tragacanthoides*, haplotype H7 had the closest relationship with haplotype GH3 in *C. gortschakovii*. The results showed that the haplotypes of the two species distributed in the western region are closely related (Figure 3e).

According to the Bayesian cluster analysis, when *K* = 3, the Deltak value is the largest. Individuals of *C. gortschakovii* are divided into one cluster, while individuals of *C. tragacanthoides* are divided into two clusters. In *C. tragacanthoides*, the populations of TI in the west and the populations of QY in the east belong to the same genetic lineage, while YHQ forms another genetic lineage (Figure 3b, d).

**FIGURE 5:**
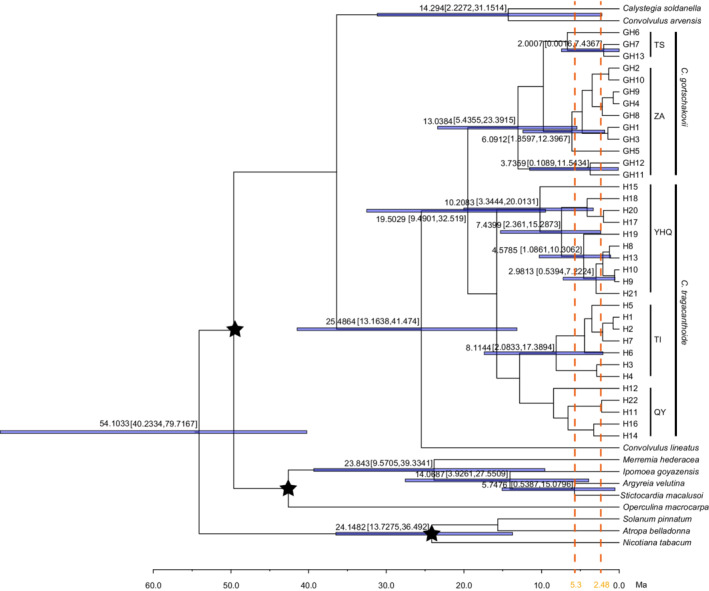
Divergence times inferred with BEAST. Numbers above branches are divergence time. The size of blue band represents 95% higher posterior densities of divergence time. YHQ, west Yin Mountains, Helan Mountains, and Qinglian Mountains; TI, Tianshan Mountains and Ili valley; QY, Qinling Mountains and east Yin Mountains; ZA, Junggar Basin and Alxa Desert; TS, Tianshan Mountains. Asterisks represent fossil calibrations.

In the phylogenetic trees, *C. tragacanthoides* and *C. gortschakovii* were also resolved as monophyletic and the phylogenetic tree was well supported (Figure 5). The phylogenetic tree was consistent with the results of the network analysis, and all haplotypes were divided into five groups. The results of molecular dating through BEAST V. 1.8.0 showed that divergence between *C. tragacanthoides* and *C. gortschakovii* occurred during the Miocene at 19.5029 Ma (95% higher posterior densities: 9.4901–32.519 Ma). The five main groups (TS, ZA, YHQ, TI, and QY) mainly diverged during the Miocene. The nearest common ancestor of all 22 haplotypes of *C. tragacanthoides* could be traced back to the Miocene at 15.7652 Ma. The most recent divergence occurred during the Pliocene and Pleistocene periods.

### 3.3 Present and past distribution modeling

**FIGURE 6:**
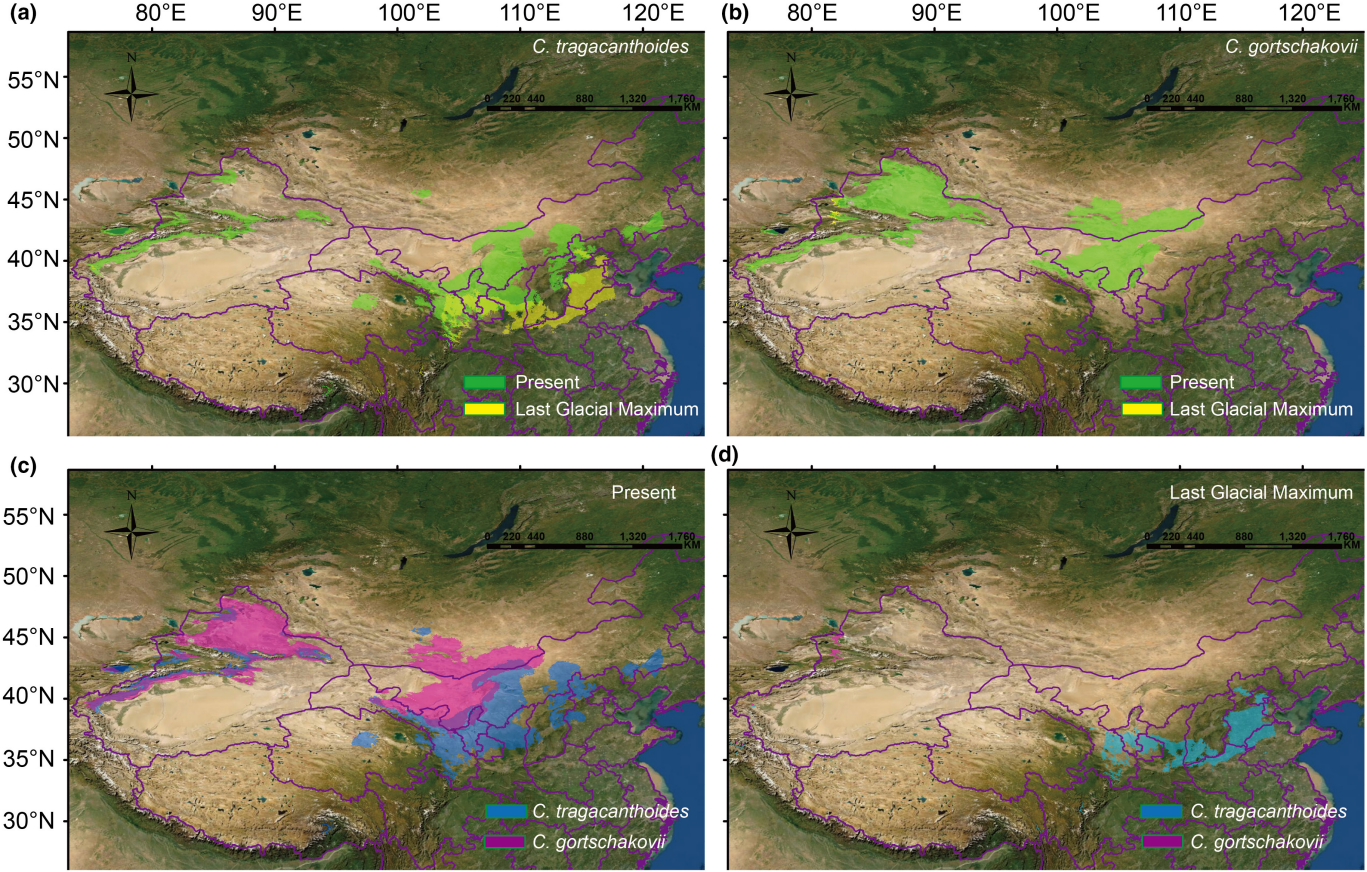
Potential species distributions of *Convolvulus tragacanthoides* and *Convolvulus gortschakovii*. (a) Compare the changes of the most suitable potential distribution area (occurrence probability >0.6) of *C. tragacanthoides* in present and Last Glacial Maximum. (b) Compare the changes of the most suitable potential distribution area (occurrence probability >0.6) of *C. gortschakovii* in present and Last Glacial Maximum. (c) Compare the changes of the most suitable potential distribution area (occurrence probability >0.6) of *C. gortschakovii* and *C. tragacanthoides* in present. (d) Compare the changes of the most suitable potential distribution area (occurrence probability >0.6) of *C. gortschakovii* and *C. tragacanthoides* in the Last Glacial Maximum.

The MAXENT model was used to simulate the geographical distribution of *C. tragacanthoides* and *C. gortschakovii* (Figure 6, Figure S3). After 10 cross‐validation, the mean values with SD of test AUC were 0.9934 ± 0.0031 and 0.9922 ± 0.004, respectively, indicating that the model had a high goodness of fit to the observation data set. For *C. tragacanthoides*, the annual mean temperature (38.3057%) and precipitation of driest month (17.8432%) explained more than half of all variation (56.1489%) observed in the distribution of *C. tragacanthoides*. During the LGM, the potential geographic distribution was significantly smaller than that during the present period. For *C. gortschakovii*, precipitation of coldest quarter (25.4588%), mean temperature coldest quarter (21.0369%), and annual precipitation (12.9671%) explained more than half of the variation (59.46%) in the distribution of the species. Compared with the present geographical distribution range, the potential distribution area in the LGM is smaller. During the mid‐Holocene period, the potential geographic distributions of the two species were closer to the present geographic distribution (Figure S3).

**FIGURE 7:**
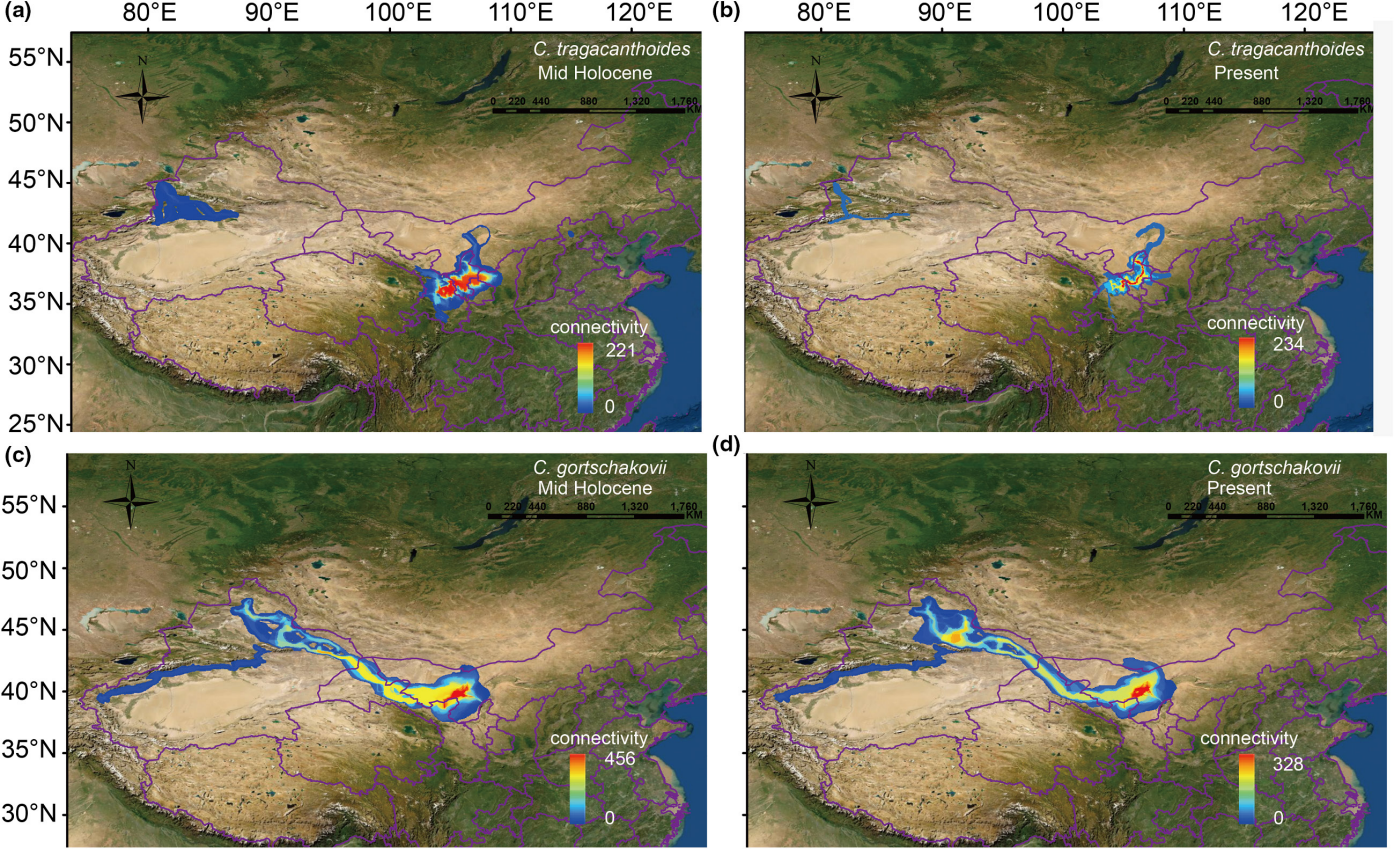
Potential dispersal corridors for *Convolvulus gortschakovii* and *Convolvulus tragacanthoides* estimated by SDMTOOLBOX. (a) Potential dispersal corridors for *C. tragacanthoides* in the mid‐Holocene. (b) Potential dispersal corridors for *C. tragacanthoides* at present. (c) Potential dispersal corridors for *C. gortschakovii* in the mid‐Holocene. (d) Potential dispersal corridors for *C. gortschakovii* at present.

The analysis of the lowest cost path showed that, in the mid‐Holocene and at present, *C. tragacanthoides* had a dispersal corridor along the TI in the western region, and a dispersal corridor along the YHQ and Qinling Mountains in the eastern region, the east and west dispersal corridors are not connected (Figure 7). During the mid‐Holocene and that of present times, *C. gortschakovii* accessed a dispersal corridor connecting its eastern and western regions and a dispersal corridor along the southern part of the Tianshan Mountains (Figure 7).

## 4 DISCUSSION

### 4.1 Phylogeographic structure of *C. tragacanthoides*

Environmental differences between the three floristic regions in China lead to the genetic differentiation of *C. tragacanthoides*. Populations of *C. tragacanthoides* in northwest China are divided into three genetic lineages, corresponding to the floristic division of seed plants in China (Wu, 1979; Wu et al., 2010). The western genetic lineage belongs to the Eurasian forest subkingdom, and the two eastern genetic lineages belong to the Sino‐Japanese floristic regions and the Asian desert flora subkingdom. The vegetation types and climates of the three floristic regions were significantly different. Therefore, *C. tragacanthoides*, which is distributed among different regions, is locally adapted, whereby a similar trend has been observed in other species such as *C. gortschakovii* (Jia & Zhang, 2021) and *Taxus wallichiana* (Gao et al., 2007). The estimation of divergence time indicated that the divergence of the three main genetic lineages of *C. tragacanthoides* occurred during the Miocene, corresponding to the time of the Tibetan Plateau uplift. The Miocene was also a period of rapid uplift in the eastern Qilian Mountains (Pang et al., 2019; Zhang et al., 2021), western Qinling Mountains (Wang et al., 2021), and Tianshan Mountains (Sun et al., 2004; Wang et al., 2010). With the uplift of the mountain, northwest China gradually became more arid. According to the analysis results of the field investigation and SDM, *C. tragacanthoides* cannot adapt to an arid climate with annual precipitation <200 mm; therefore, post the mountainous uplift events, the groups distributed on the relatively humid hillsides persisted. However, populations living in non‐uplifted areas exhibited local adaptation or extinction. This result is consistent with those of studies. The climatic and geological changes observed since the Tertiary have had a far‐reaching impact on the distribution of flora (Lu et al., 2018; Zhou et al., 2017; Zhu, 2018). Therefore, it is speculated that the genetic differentiation between YHQ, QY, and TI may be due to the gradual aridification of the northwestern region caused by the uplift of the Tibetan Plateau since the Miocene, which led to the geographical isolation of *C. tragacanthoides*. This is consistent with previous studies showing that drought caused by the Tibetan Plateau uplift has a profound impact on the differentiation and evolution of plants in northwest China (Wang & Zhang, 2022; Zhou et al., 2017). Interestingly, both the phylogenetic tree and the Bayesian clustering results support the notion that the geographically distant TI and QY are more closely related, while the geographically proximate QY and YHQ are more distantly related. We speculate that the environmental conditions of the Eurasian forest subkingdom and the Sino‐Japanese floristic regions may be more suitable for the persistence of *C. tragacanthoides*, while the environmental conditions of the Asian desert flora subkingdom are significantly different; therefore, the genetic lineages of *C. tragacanthoides* were more closely related in the Eurasian forest subkingdom and the Sino‐Japanese floristic regions.

### 4.2 Population dynamics of *C. tragacanthoides*

During the Pleistocene, the periodic cold and drought in the arid area of northwest China had a great impact on the geographical distribution of local plants. The SDM results showed that the present distribution area of the two species was significantly larger than that during the LGM (Figure 6). We also found that the distribution area during the LGM period was significantly reduced and shifted southward of *C. tragacanthoides*, when compared with the present distribution area (Figure 6). Although MDA rejected a rapid expansion, the haplotype network in the showed a star‐like distribution, centered on haplotypes H10 and H11, which was in line with the performance notion of a recent expansion (Figure 3). The neutral test of *C. tragacanthoides* in the eastern region was also suggestive of a sudden population expansion. Thus, the distribution area of *C. tragacanthoides* conformed to the pattern of general species retreating southward during the glacial period and northward during the interglacial period. In the arid area of northwest China, few plants spread from north to south during the interglacial period, and most were affected by the alternation between dry and wet conditions. During the dry glacial period, the plants of this region remained in the humid glacial refugia, and during the humid interglacial period, they dispersed, but there was no obvious north–south diffusion, such as that seen in *Atraphaxis frutescens* (Xu & Zhang, 2015), *C. gortschakovii*, *Gymnocarpos przewalskii* (Jia & Zhang, 2019; Zhang et al., 2020), *Clematis sibirica*, and *C. songorica* (Zhang et al., 2013). The origin and environmental factors surrounding species distribution may lead to different responses to environmental changes. According to the analysis of ancestral habitat reconstruction, some *C. tragacanthoides* originated in Sino‐Japanese floristic regions. Many plants distributed in the Sino‐Japanese floristic regions contracted and expanded in the north–south direction during the glacial–interglacial cycle, such as *Thuja standishii* (Worth et al., 2021) and *Vincetoxicum japonicum* (Li et al., 2016). However, *C. gortschakovii* originated in the Asian desert flora subkingdom, and like many plants of desert origin, there was no obvious north–south contraction and expansion during glacial–interglacial cycle. The landscape connectivity analysis revealed that the dispersal corridor of *C. tragacanthoides* was mainly at the edge of TI, YHQ, and QL (Figure 7). This result was consistent with the humidity adaptations exhibited by this species. Previous studies (Jia & Zhang, 2021; Zhang & Zhang, 2012) have found similar results, having shown that the relatively humid mountain foot in arid area is an important dispersal channel for desert plants.

### 4.3 Genetic variation in *C. gortschakovii* and *C. tragacanthoides*

Interspecific variation among the two species was obvious. The molecular data analysis showed that the two species did not share haplotypes and could be completely separated in the phylogenetic tree and Bayesian cluster analyses (Figures 3, 5). Similarly, according to the SDM analysis, the optimal distribution areas of the two species barely overlapped, neither at present nor during the LGM (Figure 6). These results are consistent with those of the field investigation, suggesting that the two species occupy different niches, which may be the cause of interspecies differentiation.

The present study found that the genetic differentiation and geographical isolation zones of the two species in the eastern distribution area were almost consistent with the summer monsoon limit line (200 mm precipitation line), whereby the Taihang Mountain‐Yinshan Mountain‐Helan Mountain‐Qilian Mountain form the boundary. There are great differences in altitude on both sides of the summer monsoon limit line, and ecological differences caused by complex terrain and climatic factors promote species differentiation (Tang et al., 2018). The formation of the monsoon and nonmonsoon regions is related to the uplift of the Tibetan Plateau and its nearby mountains. Due to the influence of tall mountains, the inland region situated west of the summer monsoon limit line is not significantly affected by the monsoon, with less rainfall and a dry climate, while the climate to the east of the summer monsoon limit line is relatively humid. The effect of this line on species differentiation has been demonstrated by Tang et al. (2018). The divergence time of *C. tragacanthoides* and *C. gortschakovii* was 19 Ma during the Miocene, which was consistent with the time of formation of the summer monsoon limit line. Our results suggest that the isolation of *C. tragacanthoides* and *C. gortschakovii* in the east of their distribution area might have coincided with the establishment of the summer monsoon limit line. Similarly, the uplift of the Tibetan Plateau led to the uplift of the Tianshan Mountains. The Tianshan Mountains remain relatively wet, and the low‐altitude areas around the Tianshan Mountains are gradually becoming more arid. The difference in humidity led to the divergence between *C. tragacanthoides* and *C. gortschakovii* in the west of their distribution area. Combined with the sampling survey results, the 200 mm precipitation line formed the divide between the two niches occupied by the two species. In addition, the habitat reconstruction showed that *C. tragacanthoides* originated in the east and *C. gortschakovii* originated in the west region; thus, the difference in the region of origin may also contribute toward differentiation.

The distribution and evolution of desert vegetation are closely related to many environmental factors, and the precipitation factor is one of the most important factor (Li et al., 2005; Qi et al., 2021; Xu, 2005; Zhang et al., 2017). For example, Li et al. (2005) found that there was a significant positive correlation between normalized vegetation index and precipitation, but the correlation between normalized vegetation index and temperature change was not obvious, indicating that precipitation is the main natural factor affecting vegetation change in northwest China. Similarly, Lu et al. (2018) showed that the mean divergence time of genus of angiosperm assemblages in China was more strongly correlated with annual precipitation than with annual mean temperature. Qian et al. (2020) also found that the diversity and abundance of angiosperms in China is more strongly correlated with annual precipitation than with annual average temperature, and the 500 mm precipitation line is an important dividing line of diversity and abundance. However, there are few reports on how the summer monsoon limit line (the 200 mm precipitation line) affects species distribution. In this study, from the perspective of precipitation, the impact of the formation of the summer monsoon limit line on species divergence and speciation is reported, which provides a new perspective for studying the response mechanism of species to the formation of the summer monsoon limit line and also provides a clue for predicting how desert plants respond to future environmental changes.

## AUTHOR CONTRIBUTIONS

**Shuwen Jia:** Conceptualization (equal); data curation (equal); formal analysis (equal); investigation (equal); methodology (equal); project administration (equal); resources (equal); software (equal); supervision (equal); validation (equal); visualization (equal); writing – original draft (equal); writing – review and editing (equal). **Lina Xu:** Formal analysis (equal); investigation (equal); writing – review and editing (equal). **Xiaoxiao Geng:** Software (equal). **Hong‐Xiang Zhang:** Funding acquisition (equal); resources (equal); software (equal); visualization (equal); writing – review and editing (equal).

## CONFLICT OF INTEREST

The authors declare no competing interests.

## Supporting information


Appendix S1

## Data Availability

Sequence data of northwest China *Convolvulus tragacanthoide* are available on GenBank (http://www.ncbi.nlm.nih.gov/genbank/) under accession numbers ON338045–ON338066.
