# Supplementary material for: Comparative evolutionary history of two closely related desert plant, Convolvulus tragacanthoide and Convolvulus gortschakovii (Convolvulaceae) from northwest China
Source: Ecol Evol. 2022 Sep 20;12(9):e9355. doi: 10.1002/ece3.9355 (PMC9486504; doi:10.1002/ece3.9355)

**Appendix:**

Table S1. GenBank accession numbers of outgroups used in present study.

| Species name GenBank accession numbers |
| --- |
| *Convolvulus lineatus*  MN161812 MN161823  *Convolvulus arvensis* NC054224  *Calystegia soldanella* NC060788  *Argyreia velutina* NC060787  *Atropa belladonna*  AJ316582  *Ipomoea goyazensis*  NC042938  *Merremia hederacea* NC060793  *Nicotiana tabacum*  AP019625  *Operculina macrocarpa*  KF242502  *Solanum pinnatum*  NC062490  *Stictocardia macalusoi* KF242503 |

Table S2. Climate variables used to fit niche model in *Convolvulus tragacanthoide*.

| Variable |  | Source |
| --- | --- | --- |
| BIO1 | Annual Mean Temperature (℃*10) | <http://www.worldclim.org/> |
| BIO2 | Mean Diurnal Range (Mean of monthly (max temp - min temp)) (℃*10) | <http://www.worldclim.org/> |
| BIO3 | Isothermality (BIO2/BIO7) (×100) | <http://www.worldclim.org/> |
| BIO6 | Min Temperature of Coldest Month (℃*10) | <http://www.worldclim.org/> |
| BIO7 | Temperature Annual Range (BIO5-BIO6) (℃*10) | <http://www.worldclim.org/> |
| BIO8 | Mean Temperature of Wettest Quarter (mm) | <http://www.worldclim.org/> |
| BIO12 | Annual Precipitation (mm) | <http://www.worldclim.org/> |
| BIO13 | Precipitation of Wettest Month (mm) | <http://www.worldclim.org/> |
| BIO14 | Precipitation of Driest Month (mm) | <http://www.worldclim.org/> |
| BIO17 | Precipitation of Driest Quarter (mm) | <http://www.worldclim.org/> |

Table S3. Climate variables used to fit niche model in *Convolvulus gortschakovii*.

| Variable |  | Source |
| --- | --- | --- |
| BIO2 | Mean Diurnal Range (Mean of monthly (max temp - min temp)) (℃*10) | <http://www.worldclim.org/> |
| BIO3 | Isothermality (BIO2/BIO7) (×100) | <http://www.worldclim.org/> |
| BIO4 | Temperature Seasonality (standard deviation ×100) | <http://www.worldclim.org/> |
| BIO7 | Temperature Annual Range (BIO5-BIO6) (℃*10) | <http://www.worldclim.org/> |
| BIO8 | Mean Temperature of Wettest Quarter (℃*10) | <http://www.worldclim.org/> |
| BIO10 | Mean Temperature of Warmest Quarter (℃*10) | <http://www.worldclim.org/> |
| BIO11 | Mean Temperature of Coldest Quarter (℃*10) | <http://www.worldclim.org/> |
| BIO12 | Annual Precipitation (mm) | <http://www.worldclim.org/> |
| BIO18 | Precipitation of Warmest Quarter (mm) | <http://www.worldclim.org/> |
| BIO19 | Precipitation of Coldest Quarter (mm) | <http://www.worldclim.org/> |

Table S4. The accession numbers of cpDNA sequences reported in this study.

| Sample |  | Accession number of trnT - trnY intron | Accession number of rpl14 - rpl36 region |
| --- | --- | --- | --- |
| X1 |  |  |  |
| 431 |  | ON338045 | ON338052 |
| 432 |  | ON338045 | ON338052 |
| 435 |  | ON338045 | ON338052 |
| 437 |  | ON338045 | ON338052 |
| X2 |  |  |  |
| 391 |  | ON338045 | ON338053 |
| 392 |  | ON338045 | ON338053 |
| 393 |  | ON338045 | ON338053 |
| 398 |  | ON338045 | ON338053 |
| X3 |  |  |  |
| 21 |  | ON338046 | ON338053 |
| 22 |  | ON338046 | ON338053 |
| 24 |  | ON338045 | ON338053 |
| 26 |  | ON338045 | ON338053 |
| 27 |  | ON338046 | ON338054 |
| X4 |  |  |  |
| 32 |  | ON338046 | ON338053 |
| 33 |  | ON338046 | ON338053 |
| 34 |  | ON338046 | ON338053 |
| 35 |  | ON338046 | ON338053 |
| 36 |  | ON338046 | ON338054 |
| X5 |  |  |  |
| 51 |  | ON338045 | ON338055 |
| 52 |  | ON338046 | ON338053 |
| 53 |  | ON338045 | ON338053 |
| 56 |  | ON338046 | ON338053 |
| 57 |  | ON338045 | ON338053 |
| X6 |  |  |  |
| 141 |  | ON338045 | ON338054 |
| 142 |  | ON338045 | ON338053 |
| 143 |  | ON338045 | ON338054 |
| 144 |  | ON338045 | ON338055 |
| 145 |  | ON338045 | ON338055 |
| 146 |  | ON338045 | ON338053 |
| X7 |  |  |  |
| 71 |  | ON338046 | ON338053 |
| 73 |  | ON338046 | ON338053 |
| 74 |  | ON338046 | ON338053 |
| 75 |  | ON338046 | ON338053 |
| 76 |  | ON338046 | ON338053 |
| X8 |  |  |  |
| 93 |  | ON338046 | ON338053 |
| 94 |  | ON338046 | ON338053 |
| 97 |  | ON338045 | ON338053 |
| 98 |  | ON338045 | ON338053 |
| X9 |  |  |  |
| 111 |  | ON338046 | ON338053 |
| 112 |  | ON338046 | ON338053 |
| 113 |  | ON338045 | ON338056 |
| 114 |  | ON338046 | ON338053 |
| X10 |  |  |  |
| 81 |  | ON338046 | ON338053 |
| 82 |  | ON338046 | ON338053 |
| 84 |  | ON338046 | ON338053 |
| 85 |  | ON338046 | ON338053 |
| 86 |  | ON338046 | ON338053 |
| 87 |  | ON338046 | ON338053 |
| X11 |  |  |  |
| 361 |  | ON338045 | ON338053 |
| 364 |  | ON338045 | ON338053 |
| 366 |  | ON338045 | ON338053 |
| 367 |  | ON338045 | ON338053 |
| 368 |  | ON338045 | ON338053 |
| G1 |  |  |  |
| 371 |  | ON338048 | ON338057 |
| 372 |  | ON338047 | ON338057 |
| G2 |  |  |  |
| 181 |  | ON338049 | ON338057 |
| 182 |  | ON338049 | ON338057 |
| 183 |  | ON338049 | ON338057 |
| 185 |  | ON338049 | ON338057 |
| G3 |  |  |  |
| 311 |  | ON338047 | ON338058 |
| 314 |  | ON338047 | ON338058 |
| 316 |  | ON338047 | ON338058 |
| 317 |  | ON338047 | ON338059 |
| G4 |  |  |  |
| 171 |  | ON338050 | ON338057 |
| 172 |  | ON338048 | ON338057 |
| 173 |  | ON338050 | ON338057 |
| 174 |  | ON338048 | ON338057 |
| G5 |  |  |  |
| 322 |  | ON338047 | ON338060 |
| 323 |  | ON338047 | ON338058 |
| 324 |  | ON338047 | ON338058 |
| 325 |  | ON338047 | ON338058 |
| G6 |  |  |  |
| 331 |  | ON338047 | ON338058 |
| 333 |  | ON338047 | ON338058 |
| 334 |  | ON338047 | ON338058 |
| 335 |  | ON338047 | ON338061 |
| 337 |  | ON338049 | ON338058 |
| G7 |  |  |  |
| 341 |  | ON338047 | ON338058 |
| 342 |  | ON338047 | ON338058 |
| 343 |  | ON338047 | ON338058 |
| 344 |  | ON338047 | ON338058 |
| 345 |  | ON338047 | ON338058 |
| G8 |  |  |  |
| 191 |  | ON338049 | ON338057 |
| 192 |  | ON338049 | ON338057 |
| 193 |  | ON338049 | ON338062 |
| 196 |  | ON338049 | ON338057 |
| 197 |  | ON338049 | ON338057 |
| G9 |  |  |  |
| 201 |  | ON338049 | ON338057 |
| 202 |  | ON338049 | ON338057 |
| 203 |  | ON338049 | ON338057 |
| 204 |  | ON338049 | ON338057 |
| 205 |  | ON338049 | ON338057 |
| G10 |  |  |  |
| 163 |  | ON338049 | ON338057 |
| 164 |  | ON338049 | ON338064 |
| 166 |  | ON338049 | ON338063 |
| 167 |  | ON338049 | ON338057 |
| N1 |  |  |  |
| 231 |  | ON338049 | ON338057 |
| 233 |  | ON338049 | ON338057 |
| 234 |  | ON338049 | ON338064 |
| 235 |  | ON338049 | ON338057 |
| 238 |  | ON338049 | ON338057 |
| N2 |  |  |  |
| 211 |  | ON338049 | ON338057 |
| 215 |  | ON338049 | ON338057 |
| 217 |  | ON338049 | ON338057 |
| 218 |  | ON338049 | ON338057 |
| N3 |  |  |  |
| 221 |  | ON338049 | ON338057 |
| 222 |  | ON338049 | ON338057 |
| 225 |  | ON338049 | ON338057 |
| 226 |  | ON338049 | ON338057 |
| 227 |  | ON338049 | ON338057 |
| N4 |  |  |  |
| 241 |  | ON338049 | ON338057 |
| 242 |  | ON338049 | ON338057 |
| 243 |  | ON338049 | ON338057 |
| 245 |  | ON338049 | ON338057 |
| 247 |  | ON338049 | ON338057 |
| N5 |  |  |  |
| 251 |  | ON338049 | ON338057 |
| 252 |  | ON338049 | ON338057 |
| 253 |  | ON338049 | ON338057 |
| 254 |  | ON338049 | ON338062 |
| 255 |  | ON338049 | ON338065 |
| 256 |  | ON338049 | ON338057 |
| N6 |  |  |  |
| 271 |  | ON338047 | ON338058 |
| 272 |  | ON338047 | ON338058 |
| 273 |  | ON338047 | ON338058 |
| 274 |  | ON338047 | ON338058 |
| 275 |  | ON338047 | ON338058 |
| N7 |  |  |  |
| 262 |  | ON338049 | ON338057 |
| 263 |  | ON338049 | ON338062 |
| 264 |  | ON338049 | ON338057 |
| 265 |  | ON338049 | ON338057 |
| 266 |  | ON338049 | ON338057 |
| M1 |  |  |  |
| 291 |  | ON338049 | ON338057 |
| 292 |  | ON338049 | ON338057 |
| 293 |  | ON338049 | ON338057 |
| 295 |  | ON338049 | ON338057 |
| 296 |  | ON338049 | ON338062 |
| M2 |  |  |  |
| 381 |  | ON338049 | ON338057 |
| 382 |  | ON338049 | ON338057 |
| 384 |  | ON338049 | ON338057 |
| 385 |  | ON338049 | ON338057 |
| 386 |  | ON338049 | ON338057 |
| M3 |  |  |  |
| 281 |  | ON338049 | ON338057 |
| 282 |  | ON338049 | ON338057 |
| 283 |  | ON338051 | ON338057 |
| 287 |  | ON338049 | ON338058 |
| 288 |  | ON338049 | ON338057 |
| M4 |  |  |  |
| 301 |  | ON338049 | ON338057 |
| 302 |  | ON338049 | ON338057 |
| 303 |  | ON338049 | ON338062 |
| 307 |  | ON338049 | ON338057 |
| 308 |  | ON338049 | ON338057 |
| Q1 |  |  |  |
| 351 |  | ON338049 | ON338057 |
| 352 |  | ON338049 | ON338057 |
| 353 |  | ON338049 | ON338057 |
| 356 |  | ON338049 | ON338057 |
| HB1 |  |  |  |
| 951 |  | ON338047 | ON338066 |
| 952 |  | ON338047 | ON338066 |
| 953 |  | ON338047 | ON338066 |
| 954 |  | ON338047 | ON338066 |
| 955 |  | ON338047 | ON338066 |
| HB2 |  |  |  |
| 961 |  | ON338047 | ON338066 |
| 962 |  | ON338047 | ON338066 |
| 963 |  | ON338047 | ON338066 |
| 964 |  | ON338047 | ON338066 |
| 965 |  | ON338047 | ON338066 |

Fig. S1. Distribution of sampling points of *Convolvulus tragacanthoides* and *Convolvulus gortschakovii*. Green Triangle: *Convolvulus gortschakovii.* Red circle: *Convolvulus tragacanthoides.* The background is satellite image.


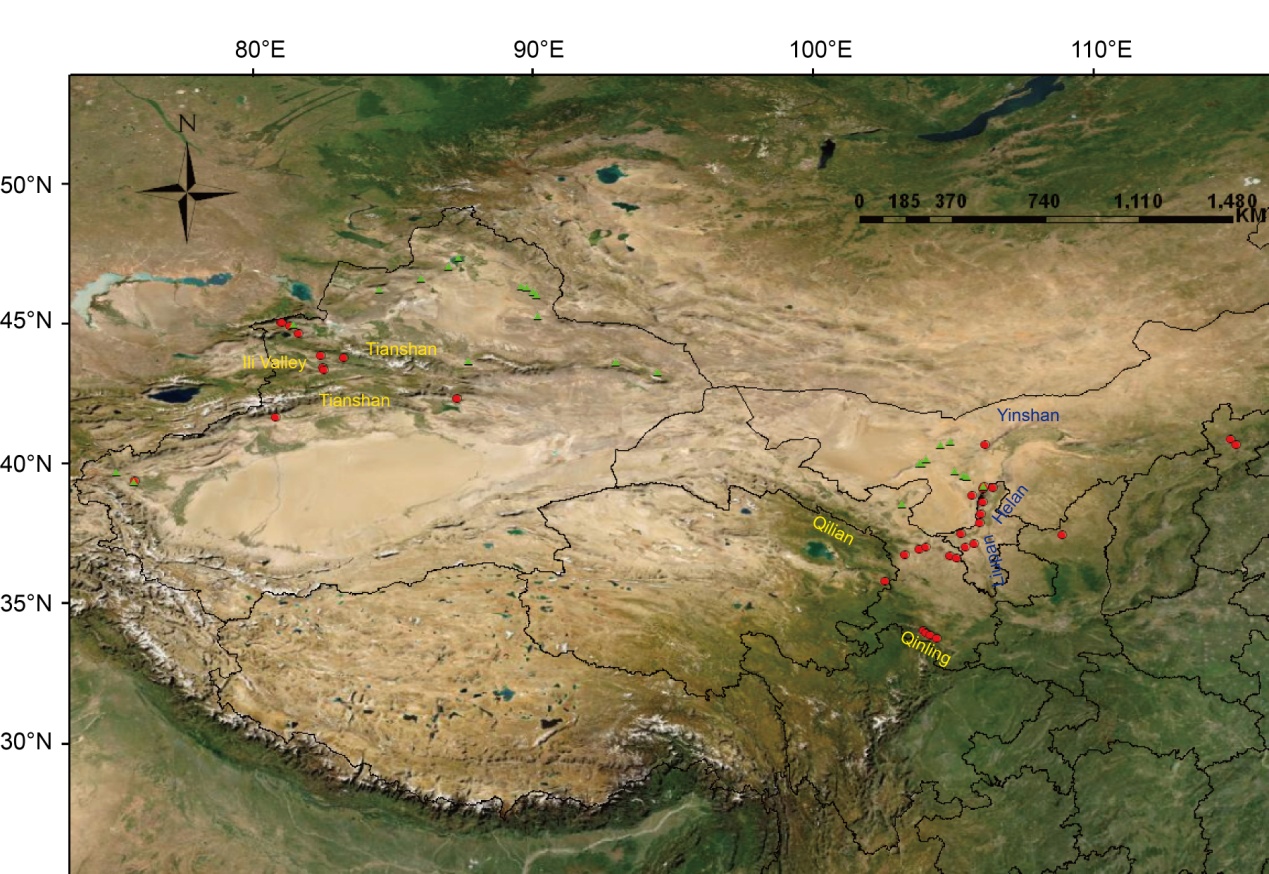


Fig. S2. Mismatch distribution analysis of *Convolvulus tragacanthoides.*


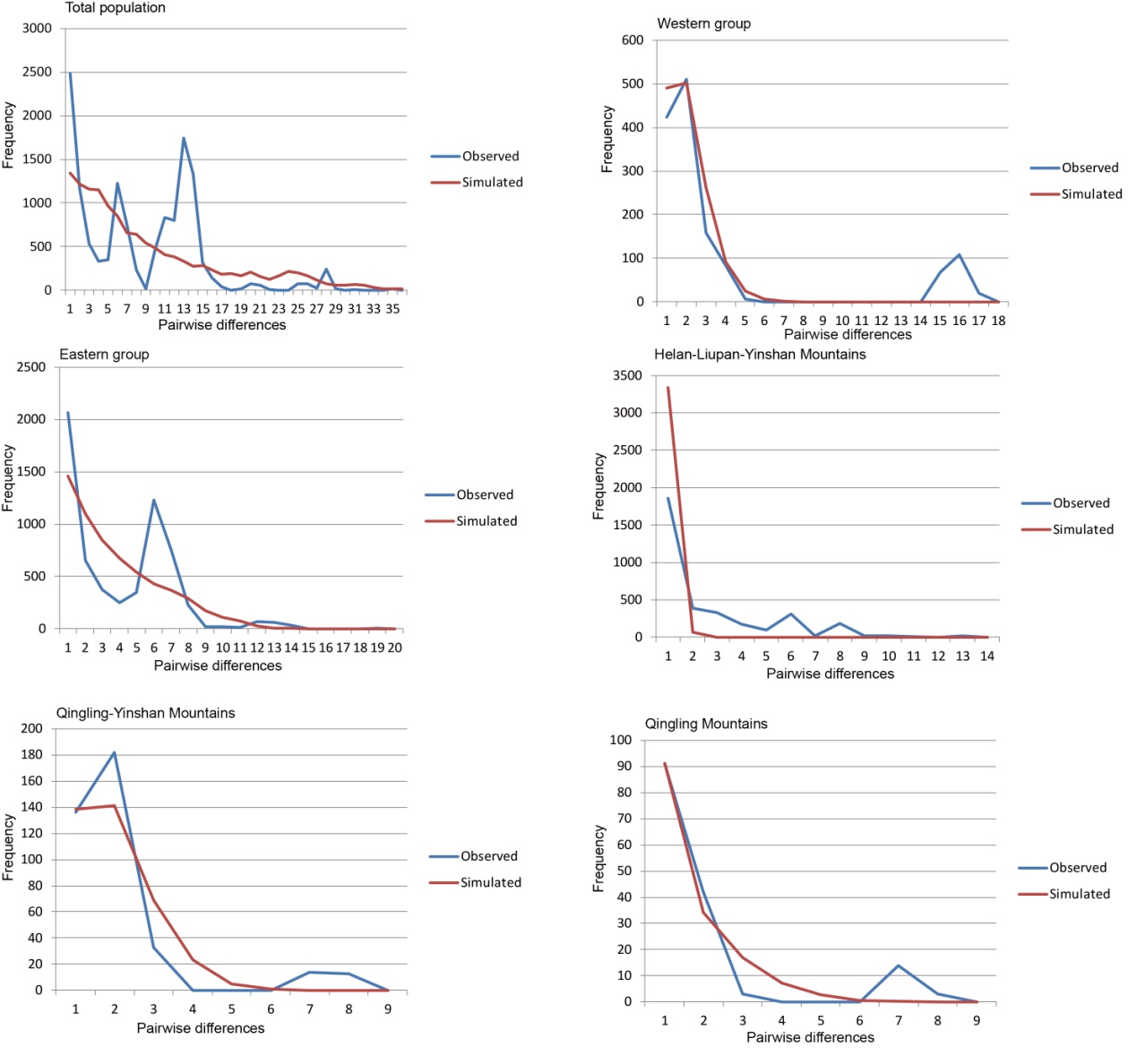


Fig. S3. Potential species distributions of *Convolvulus tragacanthoides* and *Convolvulus gortschakovii*.


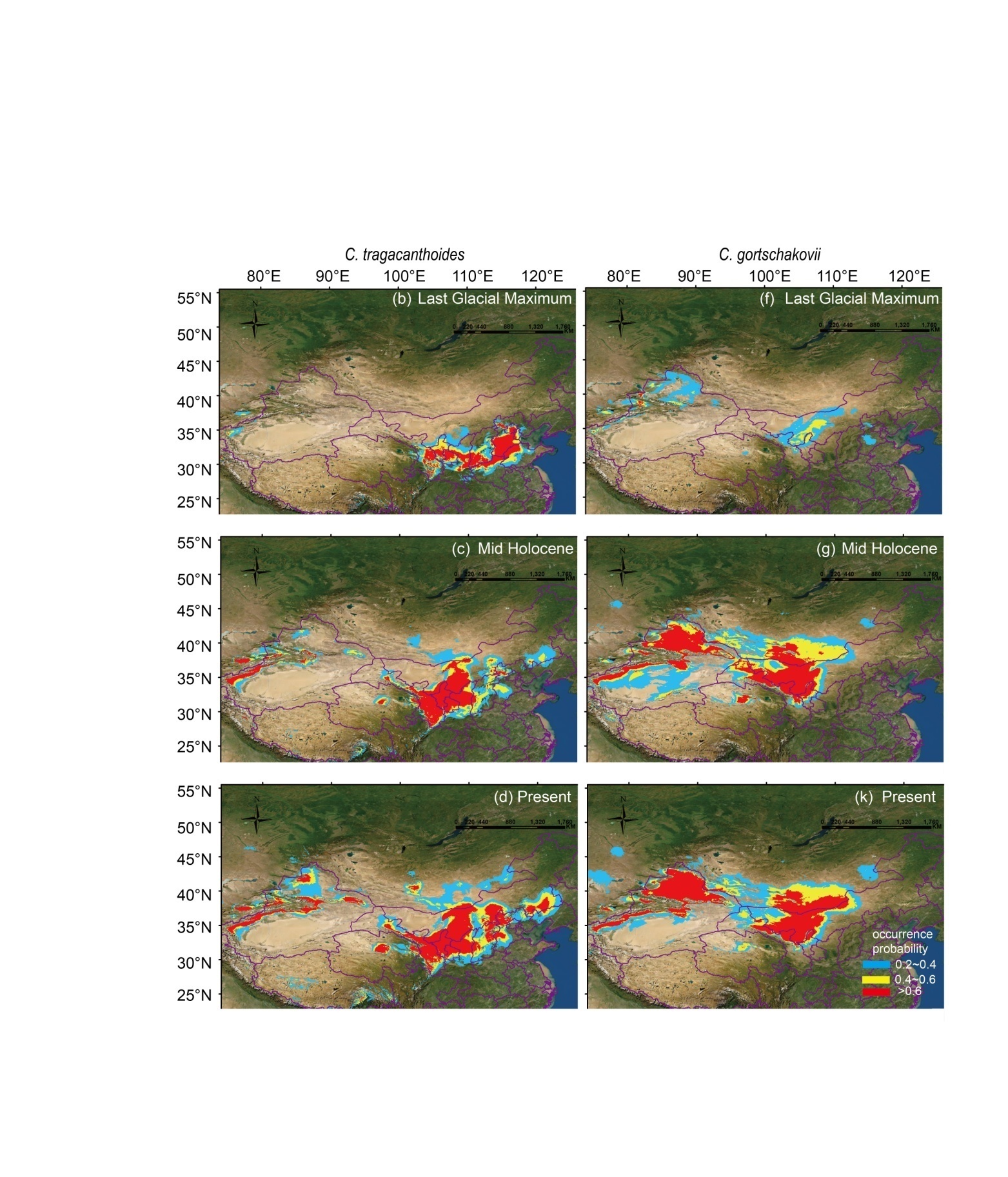

Supplement: Supplementary file 1 — Appendix S1 [file ECE3-12-e9355-s001.docx]
